# Rationale and methodology of a multicentric prospective cohort study on ‘Longitudinal Effects of Air Pollution Exposure on Adolescent Lungs (APEAL)’ in urban India: APEAL protocol

**DOI:** 10.1136/bmjopen-2025-106329

**Published:** 2025-08-12

**Authors:** Twinkle Agrawal, Harish C Phuleria, Anant Mohan, George D’Souza, Rajesh Thimmulappa, Biligere Siddaiah Jayaraj, Minu Rose Mani, Swapnali Patil, Priya Samdarshi, Amruta Nori-Sarma, Gregory Wellenius, Padukudru Anand Mahesh

**Affiliations:** 1 Department of Community Health, St John’s National Academy of Health Sciences, Bengaluru, Karnataka, India; 2 Environmental Science and Engineering Department, Indian Institute of Technology Bombay, Mumbai, Maharashtra, India; 3 Pulmonary, Critical Care and Sleep Medicine, All India Institute of Medical Sciences, New Delhi, New Delhi, India; 4 Department of Pulmonary Medicine, St John’s National Academy of Health Sciences, Bengaluru, Karnataka, India; 5 Department of Biochemistry, JSS Medical College, Mysuru, Karnataka, India; 6 Department of Respiratory Medicine, JSS Medical College, Mysuru, Karnataka, India; 7 Environmental Health, Harvard T H Chan School of Public Health, Boston, Massachusetts, USA; 8 Environmental Health, Boston University, Boston, Massachusetts, USA

**Keywords:** Pulmonary Disease, Environmental Illness, Respiratory Function Test, Adolescent, PREVENTIVE MEDICINE

## Abstract

**Abstract:**

**Introduction:**

Air pollution is a significant global health concern, with studies from the USA and Europe linking long-term exposure to respiratory issues and poor school attendance in children. While Indian cities experience much higher pollution levels, the impact on lung development in Indian children remains unclear. This study aims to assess the burden of impaired lung function in Indian children and identify key factors contributing to pollution-induced lung injury.

**Methods and analysis:**

This longitudinal, prospective cohort study is conducted in four cities categorised by particulate matter 2.5 (PM_2.5_) levels: ‘very high’ (Delhi), ‘high’ (Mumbai, Bangalore) and ‘moderate’ (Mysore). A total of 4000 participants (1000 from each city) will be included in the study. Participants will complete a structured questionnaire covering sociodemographics, asthma and allergy history (International Study of Asthma and Allergies in Childhood core questionnaire), dietary intake (24-hour recall and Food Frequency Questionnaire), Physical Activity-C Questionnaire and air pollution exposure. Spirometry and Forced Oscillation Technique will be used to assess lung function. Blood samples will be collected for identification of biomarkers to predict lung impairment. After quality checks, data will be compiled, summarising pulmonary function parameters alongside covariates and confounders. Analysis of Variance (ANOVA) will assess between-city and within-city differences in lung function.

We anticipate a higher prevalence of reduced lung function in children residing in cities with very high and high PM_2.5_ levels compared with the moderately polluted city. Findings from this study could establish normal age-appropriate lung function reference values for Indian urban children, aiding in clinical diagnosis.If a reliable biomarker for identifying children at risk of lung impairment is available, it could serve as an early predictor of poor lung health in asymptomatic children.

**Ethics and dissemination:**

The approval from individual site institutional review board (IRB) is obtained prior to initiation of the study from institutional ethics committee, St. John’s Medical College and Hospital, Bangalore; institutional ethics committee, JSS Medical College, Mysore; institute ethics committee, Indian Institute of Technology Bombay and institute ethics committee, All India Institute of Medical Sciences. Findings from this study will be disseminated through conference presentations, peer-reviewed publications and establishment of normal age-appropriate lung function reference values for children living in urban India.

**STRENGTHS AND LIMITATIONS OF THIS STUDY**

- Multicentric, prospective cohort study across India.
- Study based on pulmonary function test (PFT) values using spirometry and forced oscillation technique which is superior to symptom screening.
- Measurement of pollution levels in the study area using standard equipment to get more accurate data compared with the real time data from air quality monitoring stations.
- Development of a biomarker to predict lung diseases among children in the future.
- More children need screening for a successful PFT with an expected loss to follow-up (20%) of participants over 5 years post study initiation.

## Introduction

 The WHO recognises air pollution as the second most significant risk factor for non-communicable diseases. Major air pollutants impacting health include particulate matter (PM), carbon monoxide (CO), ozone (O₃), nitrogen dioxide (NO₂) and sulfur dioxide (SO₂). According to the WHO, in 2019 about 99% of the world’s population were living in areas with poor standards of air quality. Ambient air pollution was attributed to 4.2 million premature deaths in 2016; of these, about 300 000 were children under the age of 5 years. About 93% of all children worldwide are exposed to PM_2.5_ levels exceeding WHO air quality standards.^1^

Air pollution adversely affects lung development, leading to reduced forced expiratory volume in one second (FEV₁) and increased respiratory disease risk in adulthood.^2^ This is associated with an increase in lung disease in adulthood. Hence, understanding the effect of air pollution on lung growth and identifying those who are vulnerable is important as it may help in early intervention.^3^

Studies from high-income countries show that long-term childhood exposure to air pollution can shift population-wide lung function growth downward.^4^ A 10 µg/m³ increase in PM_2.5_ exposure has been linked to reductions in FEV₁ (61.0 mL), forced vital capacity (FVC) (54.5 mL) and peak expiratory flow (10.0 L/min).^5^ Moreover, impaired childhood lung function is associated with the development of chronic obstructive pulmonary disease (COPD) in adulthood.^6^ The FEV₁/FVC ratio at the 10th percentile has been identified as a predictor of COPD in adults, even in those without conventional airflow obstruction.^7^ The pre-bronchodilator and post-bronchodilator values of FEV1, FVC and FEV1/FVC ratio among the children could help with defining normal cut-off values, prediction of risk for development of asthma or COPD as well as prognosis of already diagnosed cases.

India’s urban population is rapidly growing,^8^ with PM_2.5_ levels showing a 2–8% annual decline in megacities. However, diurnal, seasonal and monthly variations persist due to differences in local sources and meteorology.^9^ This study aims to assess the impact of air pollution on lung development in urban Indian children across cities with varying pollution levels.

### Rationale

Air pollution is a major global health threat, with studies from the USA and Europe linking long-term exposure to respiratory issues and poor school attendance in children.^10 11^ Indian cities experience much higher pollution levels,^12^ but its impact on lung development in Indian children remains unclear. This study will assess the burden of impaired lung function in Indian children and identify critical factors contributing to pollution-induced lung injury.

Club cell protein 16 (CC16) (also known as CC10 or uteroglobin) is the predominant protein found in airway secretions, produced by CCs situated in both large and small airways.^13^ CC16 leaks into the bloodstream and the reduction in circulatory CC16 levels serves as a surrogate marker of lung damage. Circulatory levels of CC16 exhibit an inverse correlation with FEV in patients with COPD^13^ and are also reported to predict poor lung function in childhood.^14^ Chronic PM exposure causes lung damage through oxidative and inflammatory stress. Therefore, we will link CC16 levels with circulatory markers of oxidative stress (oxidised-low-density lipoprotein and total serum antioxidant capacity) and inflammation (eg, Interleukin-6) to determine the extent of lung injury in urban children associated with PM exposure levels.

Oxidative potential (OP) of PM measures its ability to generate free radicals and is linked to harmful chemical components such as polycyclic aromatic hydrocarbons and metals.^15^ Currently, air quality monitoring stations across India report PM_2.5_ as an indicator of air quality and provide health advisories as good/safe, moderate, poor, very poor and severe.^16^ The Weichenthal group^17^,^19^ studied ambient PM_2.5_ OP in cities in Ontario, Canada and reported that OP, rather than mass concentration of PM, was a key determinant of emergency hospital visits due to asthma in children. Despite PM_2.5_ being a standard air quality metric in India, no studies have explored the relationship among PM composition, OP and lung function growth in Indian children.

Lung development is also influenced by nutrition and physical activity. Diets rich in fruits, vegetables and fish are linked to better lung function,^20^ while vitamin deficiencies may impair alveolar growth.^21 22^ Physical activity benefits lung capacity, but its effects in polluted environments remain unclear.^23 24^ Hence, our objective is to conduct a longitudinal study to measure the growth of lung function among children aged 11–14 years, over a period of 5 years, residing in the major cities of India in relation to the air pollution levels. The study will also identify early blood biomarkers for a decrease in lung growth; the relationship between PM chemical composition, OP and lung function growth within and between cities in India; and the impact of nutrition and physical activity on lung function growth. Successful completion of our study will identify the burden of children with poor lung function across India and characterise critical chemical, biological and behavioural determinants of air pollution-induced lung injury.

## Methods and analysis

This study is designed as a longitudinal, prospective cohort study conducted simultaneously in four cities chosen based on the PM_2.5_ levels at the time of study initiation, to categorise them into ‘very high’ (121-250) (Delhi), ‘high’ (91-120) (Mumbai and Bangalore) and ‘moderate’ (61-90) (Mysore city) level of pollution.

### Sample size and sample size calculations

#### For the primary objective

In this study, there will be four visits (one baseline and three follow-up visits) to measure lung function growth in children aged 11–14 years across cities with varying PM_2.5_ levels (moderate, high, very high).

The sample size was calculated using the formula,^25^


$$N=\frac{2\sigma^{2}\;\left(1-\rho \right)}{T\;\times \;\delta^{2}}\times {\left(Z_{1-\frac{\alpha }{2}}+Z_{1-\beta }\right)}^{2}$$

assuming an annual lung function growth difference of 3%, a SD of 10%, a within-subject correlation of 0.7, an alpha error of 0.05 and 80% power. After adjusting for a 20% attrition rate, the study needs a sample size of 300 children per city. This results in a final sample size of 1200 children**,** ensuring sufficient power to detect meaningful differences in lung function trajectories over the four visits. To evaluate for gender-based differences, we will double the sample size to include at least 2400 children.

#### For the secondary objective

To identify risk factors associated with low lung function in children across four cities in India, using logistic regression, we aim to detect associations with an OR of 1.5 or greater. The sample size was calculated based on the following assumptions: an alpha (α) of 0.05 for two-tailed testing, a power of 80% (1-β), an outcome prevalence (P) of 30% based on previous studies of low lung function in similar populations^26^ and a risk factor prevalence (p) of 20% for the least common exposure. The formula used for calculation was


$$n=\frac{{\left(Z_{\alpha }\sqrt{P\left(1-P\right)}+Z_{\beta }\sqrt{P_{1}\left(1-P_{1}\right)}\right)}^{2}}{{\left(P_{1}-P_{2}\right)}^{2}}$$

The model will include up to 10 covariates. After adjusting for covariates and considering for four cities a total sample size of 4000 children, distributed approximately 1000 per city, will provide sufficient power to detect meaningful risk factors for low lung function with an OR of 1.5 or greater. In order to fulfil both primary and secondary objectives, the maximum sample size of 4000 (1000 per city) will be considered.

##### Inclusion and exclusion criteria

The inclusion criteria are school-going children aged ≥11 years and ≤14 years, enrolled in the school for more than 1 year, living in the same area for more than 1 year and willing to participate in the study for the entire period. Children with a history of smoking, asthma (assessed by medical history), tuberculosis or other chronic respiratory illness, chest wall deformities or any severe systemic or cardiac illness precluding performance of spirometry are to be excluded from the study.

##### Recruitment of schools

Four study sites have been chosen from all over India with representation from very high, high and moderate levels of PM_2.5_. After obtaining permission from the local administrative body to conduct the study in schools of the respective area, the list of all schools in the area will be obtained. The Global Asthma Network protocol will be used to select the schools randomly in the city. After screening the schools based on the availability of the study age group children, the eligible schools will be randomly selected with equal distribution of government, government-aided and private schools. The selected schools will be approached one by one for permission to conduct the study.

##### Impact of COVID and modification of study design

Due to the impact of COVID which started soon after the study was planned (2020), all the schools were closed due to the lockdown. Three sites (Mysore, Mumbai and Delhi) modified the protocol for a house-to-house visit for recruitment of children into the study. The Burden of Obstructive Lung Disease (BOLD) protocol was followed for a random selection of children in an administrative area of the city which has greater than 150 000 population. In a smaller city such as Mysore, all the administrative wards (n=65) were selected and 20 houses in each ward were randomly selected. In the other two large metros, an area of the city with greater than 150 000 population was selected, and the population was randomly selected, distributed equally from the administrative wards in the selected area.

##### Selection of subjects

The population of interest is school children of 11–14 years. Parents of the prospective candidates in Bangalore will be contacted with the help of school management to explain study procedures. Interested parents are to be called to the school on a date convenient for the management, parents as well as the study team. The children who are eligible to participate and whose parents consented to the study will be enrolled after explaining the procedure and signing assent forms as well as the parental consent forms. In the other three cities (Mysore, Mumbai and Delhi), the parents from the randomly selected houses with eligible children (aged 11–14 years) will be approached for their interest to participate in the study and included after obtaining parental consent and assent.

##### Study period

The recruitment of participants for the study is expected to begin by December 2021. Each participant will be followed up for 4 years. The study is expected to be concluded with analysis and publication of results by April 2026.

##### Follow-up visits

**Figure 1:**
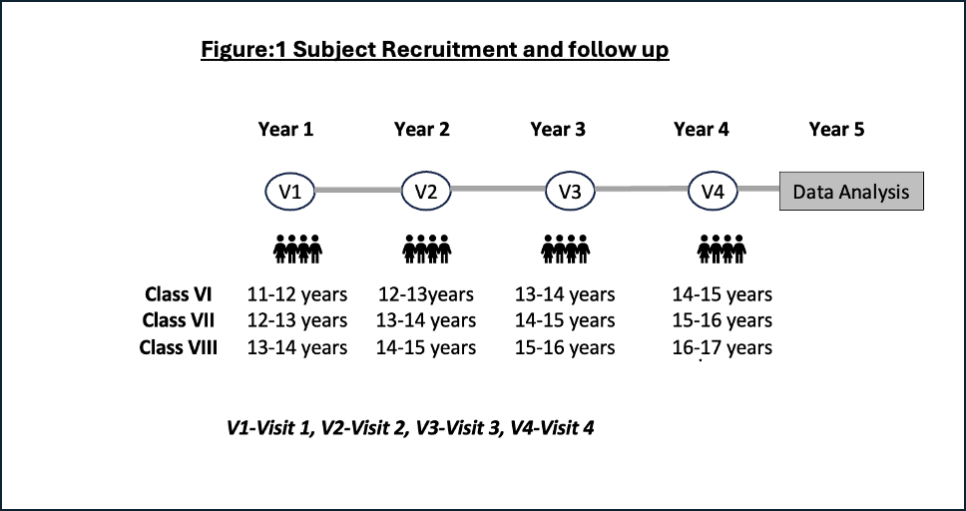
Subject recruitment and follow-up. The figure explains the target age group for recruitment and classes that they will be studying in each follow-up year (visit 1–4).

A telephonic follow-up will be done every 6 months to capture any change in demographic details, school attendance and health issues. The children will be followed up for the next 4 years (Refer figure 1). Every year the core questionnaire will be administered, pulmonary function tests and anthropometric measurements will be repeated. At the first and last visit (visit 4), blood, hair and nail samples will be collected.

##### Study tools

All participants are to be administered a structured questionnaire which contains five sections: (1) sociodemographic details; (2) core questionnaire; (3) diet-24-hour dietary recall and Food Frequency Questionnaire (FFQ); (4) Physical Activity-C questionnaire (PAQ-C); (5) air pollution exposure. The information is collected mostly from the parents, apart from a few questions which the participant can answer. The core questionnaire is a questionnaire derived from the International Study of Asthma and Allergies in Childhood study which has three modules, wheezing, rhinitis and eczema.^27^ 24-hour dietary recall will be captured for 2 days (1 weekday and 1 weekend) at each visit. FFQ will be asked only once at each visit. PAQ-C questionnaire is used to assess the physical activity of the participants. A face-validated questionnaire on air pollution exposure will be used for assessing the exposure levels of air pollution (refer: online supplemental material 1).

##### Biospecimen collection

Blood, hair and nail samples will be collected based on standard operating procedure (SOP) for sample collection at visit 1 and visit 4 (refer: online supplemental material 2).

##### Anthropometric measurements

All study personnel will be trained to measure height, weight, waist, hip, mid-upper arm circumference and triceps skinfold thickness using standardised equipment and procedures. These anthropometric measurements will be conducted for all participants at each visit.

##### Lung function test

Spirometry and forced oscillation technique (FOT) or impulse oscillometry will be done in all participants at baseline and repeated every year till 4 years. SOP for lung function tests is added as supplementary material (refer: online supplemental material 3).

### Training

All field workers will be trained before commencement of field work. The training programme will include standardised training sessions for all the sites on all aspects of the study including how to administer the questionnaire from the coordinating centre to ensure data uniformity. PFTs (spirometry and FOT), anthropometric measurements and 24-hour dietary recall training will be conducted at each of the sites itself by trained professionals.

### Patients and public involvement

Patients or the public were not involved in the design, conduct, reporting or dissemination plans of our research.

### Data management and analysis

Data will be entered into the Epicollect database as recorded in the questionnaires. After quality checks at each centre, data will be compiled, summarising pulmonary function parameters alongside covariates and confounders. The potential dropout rates would also be reported and compared against the assumed rates. Analysis of Variance (ANOVA) will assess between-city and within-city differences in lung function.

Measured parameters—including lung function, air pollution, oxidative/inflammatory markers, CC16 (a serum marker for low lung function), dietary intake and physical activity—will be summarised using appropriate descriptive statistics. Qualitative variables will be reported as counts and percentages. Baseline data will be analysed to identify time-invariant factors associated with lung function outcomes. Univariate analysis of PFT parameters will use independent t-tests or ANOVA if data are symmetric; otherwise, Box-Cox transformation or non-parametric tests will be applied. Associations between lung function and air pollution at baseline will be adjusted for confounders such as socioeconomic status, environmental factors (biomass use, household smoking, ventilation), biological factors (nutrition, genetics, pre-existing conditions), behavioural factors (physical activity, time outdoors), respiratory infections, access to healthcare, anthropometric parameters and location of school or house (urban layout, traffic), using either linear/loglinear regression or logistic regression models. Analyses will also be stratified by city, with intercity comparisons of lung function and PM_2.5_ exposure.

For longitudinal data, linear/loglinear mixed-effects or logistic mixed models will estimate the impact of high PM_2.5_ exposure on lung growth, adjusting for confounders. Linear mixed-effects models provide a flexible framework to analyse correlated data such as these. Specifically, the linear mixed-effects model is a generalisation of the standard linear model in which the data are permitted to exhibit correlation and non-constant variance. Specifically, the data for each outcome will be fit with the following model:


$$Y_{ij}=\beta_{0}+\beta_{1}\;X_{1}+...+\beta_{k}\;X_{k}+b_{i}+\varepsilon_{ij},$$

where: $Y_{ij}$ is the response of the i^th^ subject on day *j*, *β_0_, β_1_, … β_k_* represent regression coefficients for *k* fixed effects, *b_i_* represents a subject-specific effect for subject *i, ε_ij_* represents within-subject measurement error for subject *i* at time *j*.

Normally, it is assumed that the *b_i_* and *ε_ij_* terms are independent and each normally distributed with mean 0. Thus, the response for subject *i* is expected to deviate from the population mean response by a subject-specific effect (given by *b_i_*) and a within-subject measurement error (given by *ε_ij_*). Inference is based on the fixed effects given by the vector of *β*’s. Fixed effects represent the same covariates that would be included in a standard linear regression model and may include both time-invariant effects (eg, gender, age at baseline) or time-variant effects (eg, pollutant levels, temperature).

Linear mixed models are fit by restricted maximum likelihood, using standard statistical software. Specifically, we will fit these models in SAS V.9.1 (Cary, North Carolina) using the mixed procedure. Linear models assume that the response variable is approximately normally distributed. For each outcome variable, we will use descriptive statistics, histograms and other graphical techniques to assess the assumption of normality. An appropriate transformation will be applied to each outcome variable that is not normally distributed. For each outcome, the primary analysis will evaluate the association with PM_2.5_ entered as a linear continuous variable. We will control for age, gender and meteorological covariates in all models. Sensitivity analyses will be conducted to evaluate the robustness of these findings.

Stratified analyses and interaction models will explore modification effects of SES, physical activity and diet. To assess causal pathways between air pollution and lung function, mediation by oxidative stress and inflammation markers will be estimated using structural equation modelling (SEM). The association of CC16 with oxidative and inflammatory markers will be examined via regression and correlation analyses. SEM will also test the indirect effects of PM_2.5_ on CC16. A diagnostic cut-off for CC16 in low lung function will be determined using Receiver Operating Characteristic (ROC) curve analysis and reference distributions from healthy Indian adolescents.

The power of all the statistical tests for each of the estimates will be derived for the given study sample by applying appropriate power analysis methods.

### Expected outcomes

We anticipate high prevalence of children with reduced lung function living in cities with very high and high PM_2.5_ levels as compared with the moderately polluted city. Accurate interpretation of PFTs depends on the use of appropriate reference values to which the individual’s results are compared. This will establish normal age-appropriate lung function reference values for children living in urban India that could be of high value for clinical diagnosis of respiratory disease in children. If we demonstrate that the serum biochemical marker, CC16 pneumoprotein, is a reliable measure to predict, diagnose and identify susceptible children with reduced lung function, this could be translated to clinical practice to predict poor lung health in asymptomatic healthy children.

### Ethics and dissemination

The approval from individual site institutional review board (IRB) is obtained prior to initiation of the study; *institutional ethics committee, St. John’s Medical College and Hospital, Bangalore* (IEC study ref. No. 43/2021); *institutional ethics committee, JSS Medical College, Mysore* (study ref. No. JSSMC/IEC/180820/04 NCT/2020–21); *institute ethics committee, Indian Institute of Technology Bombay* (ref: proposal No. IITB-ie,C/2021/013); *institute ethics committee, All India Institute of Medical Sciences* (ref. no. IEC-359/08.05.2020, RP-59/2020). Findings from this study will be disseminated through peer-reviewed publications and will be presented at national and international conferences. The study team will also work with relevant governmental and institutional bodies to translate key findings to establish a normal age-appropriate lung function reference values for children living in urban India.

## Supplementary material

10.1136/bmjopen-2025-106329 online supplemental file 1

10.1136/bmjopen-2025-106329 online supplemental file 2

10.1136/bmjopen-2025-106329 online supplemental file 3
