# Supplementary material for: Rationale and methodology of a multicentric prospective cohort study on ‘Longitudinal Effects of Air Pollution Exposure on Adolescent Lungs (APEAL)’ in urban India: APEAL protocol
Source: BMJ Open. 2025 Aug 12;15(8):e106329. doi: 10.1136/bmjopen-2025-106329 (PMC12352163; doi:10.1136/bmjopen-2025-106329)
Supplement: online supplemental file 1 [file bmjopen-15-8-s001.pdf]

"Longitudinal effects of Air Pollution Exposures on
Lung growth and development of biomarker of lung function deficit in
Urban Children" funded by India Alliance DBT Wellcome Study"

(B) Questionnaire

Sl No.		Code Center		Ward Number				Participant Number				DD	MM	YY	Language Code	

B.1. CHILD NAME : ____

B.2. NAME OF GUARDIAN : ____

B.3. CHILD AGE : _________Years : ____

B.4. CHILD DATE OF BIRTH : ____

B. 5. CHILD GENDER : ☐ MALE ☐ FEMALE

B.6. SCHOOL NAME & ADDRESS : ____

____

____

B.7. Permanent Address : ____

____

____

B. 8. Contact Number : __________________________/__________________________

CORE QUESTIONNAIRE

**PID**						**Visit .No.**		**Date**			

C.1 What is the highest level of schooling this child's father has completed?

Primary School ☐
Middle School ☐
High School ☐
Some College (Trade/Professional/Community) ☐
Four Year College/University ☐
None ☐
Unknown ☐

C.2 What is the highest level of schooling this child's mother has completed?

Primary School ☐
Middle School ☐
High School ☐
Some College (Trade/Professional/Community) ☐
Four Year College/University ☐
None ☐
Unknown ☐

C.3 Please tell me whether this household or any person who lives in the household has/owns the following items:

READ EACH ITEM:

		YES	NO	DON'T KNOW
a.	Electricity?			
b.	Flush toilet?			
c.	Fixed telephone?			
d.	Cell telephone?			
f.	Radio?			
g.	Refrigerator?			
h.	Car?			
i.	Moped/scooter/motorcycle?			
j.	Washing machine?			
k.	Own their own home			
l.	Indoor bath or shower			
m.	Indoor tap			
n.	outdoor tap of their own			
o.	[Country to specify]			
p.	[country to specify]			

C.4. In the last year did you or any person who lives in the household ever go hungry for lack of money? Yes ☐ No ☐

C.5. How often did you or any person who lives in the household ever go hungry for lack of money? ____________________

C.6. How many*rooms* are there in your house? (Excluding kitchen and bathroom/s) ____________________

C.7. What grade /class are you in :

C.8. Family type: Joint : ☐ Nuclear : ☐ Extended : ☐

C.9. Total No. of persons in family:

C.10. Amount spent on food per month:

PID:

(D) HISTORY

D.1. Birth History:

D.1.a Weeks of Completed Gestation - (Prematurity)________________

D.1.b Normal / Caesarian Delivery ________________

D.1.c. History of IUGR Yes ☐ No ☐

D.1.d. Birth Weight ____________ Kgs

D.1.e. Any Complications during Pregnancy/ delivery Yes ☐ No ☐
If Yes, Mention Details

D.1.f. Is this child a twin? Yes ☐ No ☐

D.1.g. Does the child has siblings:
If Yes _______________ how many :________________

D.1.h. How many older brothers and/or sisters does this child have?
Number ☐

D.1.i. How many younger brothers and/or sisters does this child have?
Number ☐
(Please put 0 if there are no younger siblings).

D.1.j.1 Was this child born in(Country of Survey) India ?
Yes ☐ No ☐

D.1.j.2. If NO, what country was this child born in? Country ____________________

D.2. Immunization History:

D.2.a Is the child vaccinated as per age?

Fully Vaccinated ☐

Partially Vaccinated ☐

Not vaccinated at all ☐

D. 3. Medical History: SPIROMETRY QUESTIONNAIRE

Safety Questions

D.3.1. In the past three months have you had any surgery on your chest or Abdomen?
Yes ☐ No ☐

D.3.2. Do you have a detached retina or have you had eye surgery within The past three months? Yes ☐ No ☐

D.3.3. Have you been hospitalized for any problem within the Past month?
Yes ☐ No ☐

D.3.4. Does the participant have a resting pulse of greater than 120 beats Per minute?

Yes ☐ No ☐

D.3.5. Are you currently taking medication for tuberculosis?

Yes ☐ No ☐

D.3.6. Is there some other reason why this participant should not perform the spirometry maneuver?

Yes ☐ No ☐

If the answer to any of Questions D.3. 1 through D3.6 is "Yes", do NOT proceed with the test. Skip to the Spirometry Outcome section and mark Questions H.1 and H.2as "No", and check the second box "Participant medically excluded", for Question H.3.

D.3.7. Have you had a respiratory infection (cold) in the last three weeks?

Yes ☐ No ☐

D.3.8. Have you taken any medications for breathing in the last 24 hrs?

Yes ☐ No ☐

If yes, record name/type of medication(s) used.

1)

2)

3)

4)

If Question D.3.8. is'yes' and the medication used includes any of the types below, go to question D.3.8.a. If no, go to question D.3.8.d.

Type of Medication	Examples	
Short-acting beta-2 agonist	albuterol, salbutamol	6 hours prior to clinic visit
Anticholinergic inhaler	Atrovent, ipratropium	6 hours prior to clinic visit
Long-acting beta-2 agonist (including combination preparations that contain a LABA)	Serevent, Advair, formoterol, Symbicort, Salmeterol	12 hours prior to clinic visit
Oral beta-2 agonist	Albuterol, Levalbuterol	12 hours prior to clinic visit
Oral theophylline	Theodur	12-24 hours prior to clinic visit, depending upon preparation
Long-acting anticholinergic	Spiriva, tiotropium	24 hours prior to clinic visit

D.3.8.a. Did participant use a short acting beta agonist or anticholinergic inhaler, either alone or in combination with some other product, in the last six hours?

Yes ☐ No ☐

D.3.8.b. Did participant use a long acting beta agonist or oral beta 2 agonist, either alone or in combination with some other product, in the last 12 hours?

Yes ☐ No ☐

D.3.8.c. Did participant use an oral theophylline/ long acting anticholinergic, either alone or in combination with some other product, in the last 24 hours?

Yes ☐ No ☐

D.3.8.d. When was the last cigarette to which the child was exposed in immediate vicinity (exposure to passive Secondary smoking)?

i) ________________ hrs ago

ii) ________________ days ago

D.3.8.e. Any illnesses in past

" Requiring hospitalization Yes/ No, Specify if Yes ____________

" Requiring leave from school Yes/ No, Specify if Yes ____________

" No. of episodes of Cold/ cough/ fever in past one year -

" History of any other

Questions D.3.9.1.a. - D.3.9.s are about your breathing

D.3.9.1.a.Has this child ever had wheezing or whistling in the chest at any time in the past?

Yes ☐ No ☐

IF YOU HAVE ANSWERED "NO" PLEASE SKIP TO QUESTION D.3.9.g.

D.3.9.b. IF YOU ANSWERED "**YES**" - How old was this child when the wheezing or whistling started?

Less than 1 year	1-2	3-4	5-6	More than 6 years
☐	☐	☐	☐	☐

D.3.9.c. Has this child had wheezing or whistling in the chest **in the past12 months?**

Yes ☐ No ☐

(IF YOU HAVE ANSWERED "NO" PLEASE SKIP TO QUESTION D.3.9.g)

D.3.9.d. How many attacks of wheezing have you had in the past 12 months?

None ☐
1 to 3 ☐
4 to 12 ☐
More than 12 ☐

D.3.9.e. In the past 12 months, how often, on average, has your sleep been disturbed due to wheezing?

Never woken with wheezing ☐
Less than one night per week ☐
one or more nights per week ☐

D.3.9.f. ***In the past 12 months,*** has wheezing ever been severe enough to limit your speech to only one or two words at a time between breaths?

Yes ☐ No ☐

D.3.9.g. Have you ever had asthma?

Yes ☐ No ☐

(IF YOU HAVE ANSWERED "NO" PLEASE SKIP TO QUESTION D.3.9.q)

D.3.9.h. Was this child's asthma confirmed by a doctor?

Yes ☐ No ☐

D.3.9.i. Does this child have a written plan which tells you/him/her how to look after his/her asthma?

Yes ☐ No ☐

D.3.9.j. Has this child used any inhaled medicines e.g. puffers (use local terminology) to help his/her breathing problems at any time in the past 12 months?(when he/she did not have a cold)

Yes ☐ No ☐

(IF YOU HAVE ANSWERED "NO" PLEASE SKIP TO QUESTION D.3.9.k)

D.3.9.k. Please indicate how often you used of each of the inhaled medicines listed below in the past 12 months:

(delete the words below and put your local brand) only when needed / in short courses / every day

Asthalin/Levolin/Vwntorlin/Derihaler/Salbair	☐	☐	☐
Indacaterol……………………………………..	☐	☐	☐
Budecort/Budate/Derinile/Bunase/Budex	☐	☐	☐
Foracort/Seroflo/Budamate/Formonide/Esiflo/M Esiflo/Seretide	☐	☐	☐

D.3.9.l. Has this child used any tablets, capsules, liquids or other medicines e.g. Asthalin, Deriphylline,Montair, Singular, Montek, Telekast, Wysolone, Defcort, Wymesone, Omnacortil, Decardran, Theoasthallin that he/she swallowed to help his/her breathing at any time in the past 12 months? (when he/she did not have a cold)

Yes ☐ No ☐

(IF YOU HAVE ANSWERED "NO"PLEASE SKIP TO QUESTION :D.3.9.n.)

D.3.9.m. Please indicate how often this child used of each of the tablets, capsules, liquids or other medicines e.g. pills (use local terminology) listed below in the past 12 months:

	only when needed	/ in short courses	/ every day
Asthalin / Theoasthalin / Deriphyline	☐	☐	☐
Montair / Singular / Montek / TeletekLC Kid	☐	☐	☐
Wysolone / Defcort / Wymesone / Omnocortil / Decadran	☐	☐	☐
Advent / Augmentin / Azithral / Azee / Cehalexin	☐	☐	☐

D.3.9.n. In the past 12 months, how many times have you urgently taken this child to a doctor because of his/her breathing problems?

☐ None ☐ 1-3 ☐ 4-12 ☐ more than12

D.3.9.o. In the past 12 months, how many times have you urgently taken this child to an Emergency Department, without being admitted to hospital, because of his/her breathing problems?

☐ None ☐ 1-3 ☐ 4-12 ☐ more than12

D.3.9.p. In the past 12 months, how many times has this child been admitted to hospital because of his/her breathing problems

☐ None ☐ 1 ☐ 2 ☐ more than2

D.3.9.q. In the past 12 months, how many days (or part days) of school has this child missed because of his/her breathing problems?

☐ None ☐ 1-3 days ☐ 4-12 days ☐ more than 12 days

D.3.9.r. In the past 12 months, has this child's chest sounded wheezy during or after exercise?

Yes ☐ No ☐

D.3.9.s. In the past12 months, has this child had a dry cough at night apart from a cough associated with a cold or chest infection?

Yes ☐ No ☐

Questions D.3.10.a-D.3.12.are about nose problems which occurred when this child did not have a cold or the flu

D.3.10.a. Has this child ever had a problem with sneezing, or a runny or blocked nose when he/she DID NOT have a cold or the flu?

Yes ☐ No ☐

IF YOU HAVE ANSWERED "NO" PLEASE SKIP TO QUESTION D.3.10.c

D.3.10.b. IF YOU ANSWERED "YES" - How old was this child when the nose problem started?

Less than 1year	1-2	3-4	5-6	More than 6 years
☐	☐	☐	☐	☐

D.3.10.c In the past 12 months, has this child had a problem with sneezing or a runny, or blocked nose when he/she DID NOT have a cold or the flu?

Yes ☐ No ☐

IF YOU HAVE ANSWERED "NO"PLEASE SKIP TO QUESTION D.3.11.

D.3.10.d. In the past 12 months, has this child's nose problem been accompanied by an itchy nose?

Yes ☐ No ☐

D.3.10.e. In the past 12 months, has this child's nose problem been accompanied by itchy-watery eyes?

Yes ☐ No ☐

D.3.10.f. In the past 12 months, how much did this child's nose problem interfere with his/her daily activities?:

Not at all ☐
A little ☐
A moderate amount ☐
A lot ☐

D.3.11.a Has this child ever had hay fever? (include local names for hay fever such as allergic rhinitis)

Yes ☐ No ☐

IF YOU HAVE ANSWERED "NO" PLEASE SKIP TO QUESTION D.3.12.

D.3.11.b Was this child's hay fever confirmed by a doctor?

Yes ☐ No ☐

Questions D.3.12.a - D.3.12.h. are about this child's skin

D.3.12.a Has this child ever had an itchy rash which was coming and going for at least six months?

Yes ☐ No ☐

IF YOU HAVE ANSWERED "NO" PLEASE SKIP TO QUESTION D.3.12.g.

D.3.12.b. Has this child had this itchy rash at any time in the past 12 months?

Yes ☐ No ☐

IF YOU HAVE ANSWERED "NO" PLEASE SKIP TO QUESTIOND.3.12.g.

D.3.12.c. Has this child's itchy rash at any time affected any of the following places: the folds of the elbows, behind the knees, in front of the ankles, under the buttocks, or around the neck, ears or eyes?

Yes ☐ No ☐

D.3.12.d. At what age did this child's itchy rash first occur?

Under 2 years Age ☐

2-4 years Age ☐

5 or more ☐

D.3.12.e. Has this child's rash cleared completely at any time during the past 12 months?

Yes ☐ No ☐

D.3.12.f. In the past 12 months,how often, on average, has this child been kept awake at night by this itchy rash?

Never in the past 12 months ☐

Less than one night per week ☐

One or more night per week ☐

D.3.12.g. Has this child ever had eczema?

Yes ☐ No ☐

IF YOU HAVE ANSWERED "NO" PLEASE SKIP TO QUESTIOND.3.13

D.3.12.h. Was this child's eczema confirmed by a doctor?

Yes ☐ No ☐

D.3.13. Has this child ever been diagnosed with pneumonia or bronchopneumonia?

Yes ☐ No ☐

D.4. Family History

D.4.1. Is there a family History of Asthma?

Yes ☐ No ☐

D.4.2 Is there a family History of COPD?

Yes ☐ No ☐

D.4.3 Is there a family History of any other illness? Yes ☐ No ☐

If Yes, Please specify:____

Question E.1. is optional:

E.1. Record the exhaled carbon monoxide measurement ____________ppm (before performing spirometry)

PID:

(F) ANTHROPOMETRIC MEASUREMENTS

F.1. Height in Cms ....

F.2. Weight in Kgs ....

F.3. BMI ....

F.4. Skin Fold thickness : ....

F.5. MUAC ....

F.6. Hip Circumference: ....

F.7. Waist Circumference: ....

F.8. Any Drastic Weight Change since last 6 months? ....

(G) General Condition	G.1. Built<br>G.2 Pulse<br>G.3. BP<br>G.4. Temperature<br>G.5. Clubbing<br>G.6.Lymph nodes Inspection/ Palpation		
	**Physical signs**	**Possible Nutrition - related causes**	Tick if Present
G.7.Hair	Dull, dry, thin, sparse, color change, easily plucked, depigmentation, loss of shine.	Protein, energy deficient. Zinc /manganese/ copper deficiency	
G-8 Eyes	Yellowish lump,	Hyperlipidemia	
	Pale eyes,	Iron deficiency	
	Grey spots, dry membranes,	Vitamin A deficiency	
	Redness/fissures of eyelids,	Riboflavin deficiency	
	Paralysis of ocular muscles	Thiamin, Phosphorus deficiency	

G.9. Lips	Redness & swelling of mouth, Angular fissures, scars at corner of mouth	B3, B2, pyridoxine deficiencies	
G.10. Gums	Spongy, swollen, bleed / bruise easily, redness	Vitamin C	
G.11 Tongue	Smooth with papillae (small projection)	B-complex deficiencies, iron, protein deficiencies	
	Glossitis (magenta or scarlet/raw)	B-complex deficiencies, Deficiencies	
G.12. Taste & smell	Sense of taste and smell diminished	Zinc deficiency	
	Pallor	Iron Deficiency	
	Hyper pigmentation	Niacin deficiency	
G.13.Face	Scaling of skin around nostrils	Vitamin A, zinc, EFA, B2, pyridoxine Deficiencies	
G.14.Nails	Fragility, banding	Protein deficiency	
	Spoon- shaped	Iron deficiency	
	Slow wound healing	Zinc deficiency	
G.15. Skin	Psoriasis, scaliness<br>Black & blue marks due to skin bleeding	Biotin deficiency	
	Dryness, mosaic, sandpaper feel, flakiness	Vitamin A deficiency /excess, Niacin deficiency	
	Cutaneous flushing	Niacin deficiency	
G.16. Examination of URT	NOSE,		
	Post Pharyngeal Wall		
	Tonsils,		
	Ear		
	Sinuses		
G.17 Systemic Examination - Respiratory system	**Examination :**<br>Shape of Chest<br>Kyphosis<br>Scoliosis<br>Chest Deformity<br>Respiratory Rate<br>Rhythm<br>Auscultation<br>SPO2		
G.18.Systemic Examination - Abdomen	Inspection<br>Palpation - Pain/ tenderness<br>Auscultation		
G.19. Systemic Examination - CVS	Inspection<br>Palpation<br>Auscultation		

(H) Spirometry Outcome

H.1. Acceptable pre-bronchodilator test completed?

Yes ☐ No ☐

H.2. Acceptable post-bronchodilator test completed?

Yes ☐ No ☐

H.3. Unable to obtain satisfactory spirometry (check one)

The participant did not understand instructions ☐
The participant was medically excluded ☐
The participant was unable to physically cooperate ☐
The participant refused ☐

H.4 Were any adverse events related to the spirometry maneuver observed by the evaluator?

Yes ☐ No ☐

If yes, please briefly describe event:

H5. If the participant had a condition that would affect the result of their spirometry test (e.g., kyphosis, missing limbs, etc.) note that condition here.

I.1 What kind of floor covering is or was there this child's bed room at the following times ***(tick as many as are applicable)***

Type something…

		Never	At this time	During the first year of this child	At some other time
I.1.1	Wall to wall carpet	☐	☐	☐	☐
I.1.2.	Smooth floor (vinyl/linoleum, tiles, wood, concrete, etc.,) <u>without</u> a rug	☐	☐	☐	☐
I.1.3	Smooth floor (vinyl/linoleum, tiles, wood, concrete, etc.,) <u>with</u> a rug	☐	☐	☐	☐
I.1.4	No covering - soil or dirt	☐	☐	☐	☐

I.2. Have you made any changes in your home to prevent the symptoms of allergies or asthma or breathing problems in this child?

Yes ☐ No ☐ Not applicable ☐

I.3. In the past 12 months, have you had a cat in your home?:

Yes ☐ No ☐

I.4. In the past 12 months, have you had a dog in your home?:

Yes ☐ No ☐

I.5. **In the past 12 months,** how often, on average, have you given this child paracetamol *(use local terminology e.g. Acetaminophen, Panadol, Pamol, Tylenol)* for fever?

Never ☐
At least once a year ☐
At least once a month ☐

Dear Parent,

Nutrition is a very important part of our program in order to plan an appropriate nutrition, education activities and menu to meet your child's need. We need to know your child's eating patterns. This information also helps us to obtain an overview of the eating habits of young children as a group. Kindly take time to fill out the questioners carefully.

PID:

(J) Dietary pattern and food behavior

J.1. Type of diet consumption

a) Vegetarian b) Lacto-vegetarian
c) Ovavegetarian d) Non-vegetarian

J.2. How many meals does your child eat per day?
a) 4 meals b) 3 meals c) 2 meals

J.3. Is your child a "picky eater" ?

a) Yes b) NO

J.4. a Does your child skip meals ?

a) Yes b) NO

J.4. b, If Yes how often?

a) Daily b)Weekly c)once in a while

J.5. Does your child eat the lunch which is carried to school?

a) Yes b) NO

J.6. Does your child drinks milk regularly?

a) Yes b) NO

J.7. Does your child constantly complain of being hungry?

a) Yes b) NO

J.8. When is your child most hungry?

a) Morning b) Noon c)Evening d)Night

J.9. Your child's most favorite foods?

____

____

J.10. Your child's most disliked foods?

____

J.11. Is your child is allergic to any foods? Yes: ☐ No: ☐
If Yes, type of food :

J.12. How many times a day does your child eat snacks?
a) Once: ☐ b)Twice: ☐ c) Thrice: ☐ d)Mostfrequently ☐ e) Occasionally ☐

J.13. What type of food does your child eats for snacks?

a) Cookies ☐ b) Chips ☐ c) Cereals ☐ d) Vegetables ☐
e) Bakerystuffs ☐ f) puddings ☐ g) anyother, plz mention: ☐
h) Soft drinks ☐ i) Tea ☐ j) Coffee ☐ k) Processed Cheese ☐
l) Maggie noodles ☐ m) Ketchups ☐ n) Jams ☐

J.14. Do you offer food as a reward? Yes ☐ No ☐
If yes what foods ? ____

J.15. How is your child's appetite?

a) Good ☐ b) Very good ☐ c) Low ☐

J.16. Does your child have any food carving or addiction, like :

Sugar based ☐ Bread based ☐ Ice-creams ☐ No ☐

J.17. Is your child is having problem with

a)Poor appetite ☐ b) Food texture ☐ c) Chewing food ☐
d)Swallowing food ☐ e) None of these ☐

J.18. Does your child eat things that are not usually considered as foods?

Paste ☐ Dirt ☐ Paper ☐ Chalk ☐ Ice ☐ No ☐

J.19. Does your child have any food intolerance as you know? Yes ☐ No ☐

If yes, Plz list them: ____

____

J.20. How often does your child have a bowel movement?

a) Once ☐ b) Twice ☐ c) Thrice or more often ☐

J.21. Is your child taking a vitamin or any food supplements? Yes ☐ No ☐

If yes, what kind : ____

J.22. Does your child have any diet-related health problems ?

Diabetes ☐ Allergies ☐ any others ☐ No ☐

J.23. How much water does your child normally drink/per day?

____

J.24. Source of Water Supply at your Home?

BMC ☐ Bore-Wells ☐
If Bore-Well then ask **Q.25 Other wise next Question**

J.25. Do you use a water filter? Yes ☐ No ☐

J. 26. How many hours does the child sleep every day ? ______________________

J.27. Does your child have regular menstrual cycle? Yes ☐ No ☐ NA ☐

J.28. Do you have any other things to mention that you like? If Yes plz, describe:

DATE:

PID:

(K) 24 HOUR DIET RECALL - I

Meal timing	Food Item	Amount (in terms of standard cup size, tea spoon table spoon, and glass size)
Early morning K1		
Break fast K2		
Mid-morning K3		
Lunch K.4.		
Evening K.5.		
Dinner K.6.		
Bed time K.7.		

K.8. Fluid In-take: ________________________________

DATE:

PID:

(K) 24 HOUR DIET RECALL - I

Meal timing	Food Item	Amount (in terms of standard cup size, tea spoon table spoon, and glass size)
Early morning K1		
Break fast K2		
Mid-morning K3		
Lunch K.4.		
Evening K.5.		
Dinner K.6.		
Bed time K.7.		

K.8. Fluid In-take: ___________________________

DATE:

PID:

(L) FOOD FREQUENCY								
Food group		Frequency						
	Daily	Weekly (L2)				Monthly	Occasionally	Never
	(L1)	once	twice	thrice	weekends	(L3)	(L4)	(L5)
**Cereals & cereal pdts**								
WhiteRice								
Brownrice								
Ragi								
Wheat								
Oats								
Corn								
Jowar								
Cornflakes/Muesli/								
Extrudedproducts								
**Pulses & legumes**								
Red gram dhal								
Black gram								
Bengal gram								
Green gram								
Peas								
Soya bean /								
flour/nuggets								
Channa dal								
Cowpea								
Kidney beans								
**Green leafy vegetables**								
Spinach								
Amaranth								
Shepu								
Coriander leaves								
Curry leaves								
Doddapatre								
Agathi								
Drumstick leaves								
Fenugreek leaves								
**Roots & tubers**								
Carrot								
Onion								
Garlic								
Potato								
Sweetpotato								
Yam								
Radish								
Beetroot								

(L) FOOD FREQUENCY								
Food group	Daily (L1)	Frequency				Monthly (L3)	Occasionally (L4)	Never (L5)
		Weekly (L2)						
		once	twice	thrice	weekends			
**Other vegetables** Cabbage								
Capsicum								
Clusterbeans								
Brinjal								
Fieldbeans								
Tinda								
KnolKhol								
Bitter gourd								
Bottlegourd								
Ash gourd								
Pumpkin								
**Fruits**								
Apple								
Guava								
Orange								
Sweetlime								
Lemon								
Papaya								
Amla								
Banana								
Pomegranate								
Grapes,red								
Grapes,White								
Watermelon								
Muskmelon								
Chickoo								
Jackfruit								
Tomato								
Strawberry								
Jamboofruit								
Mango								
**Milk and milk products**								
Whole milk								
Skimmed milk								
Curds								
Paneer								
Cheese								
Khova								

(L) FOOD FREQUENCY								
Food group	Daily	Frequency				Monthly	Occasionally	Never
		Weekly (L2)						
	(L1)	once	twice	thrice	weekends	(L3)	(L4)	(L5)
Egg								
**Fats,oils and dry fruits**								
Ghee								
Butter								
Sunflower oil								
Groundnutoil								
Coconutoil								
Ricebranoil								
Oliveoil								
Vanaspathi								
Flaxseedoil								
Cashew								
Badam								
Raisins								
Figs								
Ifnon -veg								
Poultry								
Mutton								
Pork								
Beef								
Fish								
Spices								
Turmeric								
Pepper								
Junk foods								

PID:

M. Physical Activity Questionnaire (Elementary School)

Name:__________________________ Age:_______________

Sex: M________ F________ Grade:______________

Teacher:________________________

We are trying to find out about your child's level of physical activity from ***the last 7 days*** (in the last week). This includes sports or dances that make your child sweat or make your child's legs feel tired, or games that make your child breathe hard, like tag, skipping, running, climbing, and others.

Remember:

1. There are no right and wrong answers — this is not a test.
2. Please answer all the questions as honestly and accurately as you can — this is very important.

M.1. Physical activity in your child's spare time: Have your child done any of the following activities in the past 7 days (last week)? If yes, how many times? (Mark only one circle per row)

Sl.No		No	1-2	3-4	5-6	7 times or more
M.1.1	Skipping					
M.1.2	Rowing/canoeing					
M.1.3	In-line skating					
M.1.4	Tag					
M.1.5	Walking for exercise					
M.1.6	Bicycling					
M.1.7	Jogging or running					
M.1.8	Aerobics					
M.1.9	Swimming					
M.1.10	Baseball, softball					
M.1.11	Dance					
M.1.12	Football					
M.1.13	Badminton					
M.1.14	Skateboarding					
M.1.15	Soccer					
M.1.16	Street hockey					
M.1.17	Volleyball					
M.1.18	Floor hockey					
M.1.19	Basketball					
M.1.20	Ice skating					
M.1.21	Cross-country skiing					
M.1.22	Ice hockey/ringette					
M.1.23	Other: Cricket					

M.2. In the last 7 days, during the physical education (PE) classes, how often were your child very active (playing hard, running, jumping, throwing)? (Check one only.)

- My child don't do PE .... ☐
- Hardly ever………………………………………… ☐
- Sometimes.. ☐
- Quite often .... ☐
- Always .... ☐

M.3. In the last 7 days, what did your child do most of the time *at **Recess***? (Check one)

- Sat down (talking, reading, doing school work) ☐
- Stood around or walked around........................ ☐
- Ran or played a little bit................................... ☐
- Ran around and played quite a bit .... ☐
- Ran and played hard most of the time .... ☐

M.4. In the last 7 days, what did your child normally **do *at lunch*** (besides eating lunch)? (Check one only.)

- Sat down (talking, reading, doing school work)......... ☐
- Stood around or walked around .... ☐
- Ran or played a little bit... ☐
- Ran around and played quite a bit .... ☐
- Ran and played hard most of the time .... ☐

M.5. In the last 7 days, on how many days ***right after school***, did your child do sports, dance, or play games in which he/she were very active? (Check one only.)

- None .... ☐
- 1 time last week .... ☐
- 2 or 3 times last week .... ☐
- 5 times last week .... ☐
- 5 times last week .... ☐

M.6. In the last 7 days, on **how many *evenings*** did your child do sports, dance, or play games in which he/she were very active? (Check one only.)

- None .... ☐
- 1 time last week .... ☐
- 2 or 3 times last week .... ☐
- 4 or 5 last week .... ☐
- 6 or 7 times last week .... ☐

M.7. ***On the last weekend,*** how many times did your child do sports, dance, or play games in which he/she were very active? (Check one only.)

- None .... ☐
- 1 time.. ☐
- 2 – 3 times.. ☐
- 4 – 5 times.. ☐
- 6 or more times .... ☐

M.8. Which *one* of the following describes your child **best for the last 7 days**? Read *all five* statements before deciding on the *one* answer that describes your child.

A. All or most of his/her free time was spent doing things that involve little physical effort ☐

B. He/She sometimes (1 — 2 times last week) did physical things in his/her free time (e.g. played sports, went running, swimming, bike riding, did aerobics)....................... ☐

C. He/She often (3 — 4 times last week) did physical things in his/her free time................... ☐

D. He/She quite often (5 — 6 times last week) did physical things in his/her free time.......... ☐

E. He/She very often (7 or more times last week) did physical things in his/her free time...... ☐

M.9. Mark how often your child did physical activity (like playing sports, games, doing dance, or any other physical activity) for each day last week.

	None	little bit	Medium	Often	Very often
Monday					
Tuesday					
Wednesday					
Thursday					
Friday					
Saturday					
Sunday					

M.10. Were your child sick last week, or did anything prevent your child from doing normal physical activities? (Check one.)

Yes .... ☐
No .... ☐

If yes, what prevented him/her?___________________________________

M.11. *Does anytime your child felt severe breathlessness after physical activity?*

Yes .... ☐
No .... ☐

PID:

Section - N : AIRPOLLUTION & EXPOSURE QUESTIONNAIRE

N.1 Screening Questions

N.1.1 How long have you been living in this home?

☐ <1 year ☐ >1 year

N.1.2 Do you have balcony in the house?

☐ Yes ☐ No

If yes, ask Q N.1.3,else skip to QN.2.1

N.1.3 Does the balcony face towards road side?

☐ Yes ☐ No

N.2 Built Environment

N.2.1 When was your building constructed?

☐ <5 year ☐ 5-10 years ☐ 10-15 years

☐ 15years ☐ Don't know

N.2.2 What type of neighborhood do you live? (to be filled by the surveyor)

☐ Slum ☐ Nonslum

N.2.3 Which best describes the building in which you live?

☐ High rise building (more than 2 floor)

☐ one family house detached from any other house

☐ Row houses ☐ single story ☐ double storey ☐ other………………

If highrise building, then ask QN.2.4,else skip to QN.2.5

N.2.4 On which floor do you live? (to be filled by the surveyor) ______________

N.2.5 What is the floor made of ? (to be filled by the surveyor)

☐ Cement ☐ Mosaic/Tiles ☐ Wooden ☐ Carpet covered ☐ Other

N.3 Ventilation characteristics of house hold

N.3.1 How many door ways do you have in your home?

N.3.2 How many windows do you have in your home?

a. In living room?_____________

b. In bed room?_______________

N.3.3 If you have windows/doors how often do you keep the windows open every-day?

☐ All the time ☐ Only during day time

☐ Only during night time ☐ Few hours a day

N.3.4 In which of the seasons do you keep the windows open?

☐ Summer ☐ Monsoon ☐ Winter ☐ All seasons

N.3.5 Does your living room face a street/ road?

☐ Yes ☐ No

N.3.6 Does your bedroom face a street/road?

☐ Yes ☐ No

N.3.7 Do you have air condition (AC) in your home?

☐ Yes ☐ No

N.3.8 How often is air conditioning used to cool this home in the summer months?

	No AC	< 14 hrs /week (2hrs per day)	e" 14 hrs /week and d" 56hrs/week	>56 hrs /week (8 hrs per day)
In the living room				
In the bedroom				

N.3.9 How much are you annoyed by outdoor air pollution (from traffic, industry, etc.) if you keep the windows open? (*Provide your rating on ascale of 0-10 with 0= no disturbance at all, and 10 = intolerable disturbance)*

………………

N.3.10 Are there any major outdoor sources of air pollution within vicinity/100 meters of your house hold?

☐ Yes ☐ No

If yes then ask QN.3.11 else skip to QN.3.12

N.3.11 What is the source of the air pollution?

☐ Traffic/vehicular ☐ Industry ☐ Construction ☐ Other

N.3.12 How often do truck spass through the street where you live, on week days?

Never ☐
Seldom ☐
Frequently through the day ☐
Almost the whole day ☐

N.3.13 Have you installed any air pollution filter at home

☐ Yes ☐ No

If Yes,ask Q N.3.14 or elseskipto Q N.4.1

N.3.14 How frequently do you use the air pollution filter at home?

☐ All the time ☐ Only during day time
☐ Only during night time ☐ Few hours a day

N.4 Typical sources of air pollutants in indoor environments

N.4.1 Does some one smoke cigarette/beedies in house?

☐ Yes ☐ No

If yes then ask QN4.2 & N 4.3 or else skip to QN. 4.4

N.4.2 How many people living in this home smoke inside the home?

Number ? ☐

N. 4.3 If the answer is one person or more please identify how many cigarettes/ beedies per day on average, are smoked inside this home by:-

Person A	
Person B	
Person C	
Person D	

N.4.4 Do you burn 'incense'/'dhoop'/'diya'/'candles 'inhouse?

☐ Yes ☐ No

If yes then ask QN 4.5 or else skip to QN.4.6

N.4.5 What is number of 'incense'/'dhoop'/'diya'/ 'candles 'burnt in the house in 24hr?

Substance burnt per day	Number
Incense	
Dhoop	
Diya	
Candles	

N.4.5 Do you burn any material (eg. mosquito coils/coconut shell/agarbatti) to ward off mosquitoes in house?

Yes, through out the year ☐ Yes, during rainy season only ☐ No ☐

N.4.6 How often do you sweep/vacuum your floor?

☐ Once daily ☐ Twice daily ☐ More than two times daily

☐ Alternate days ☐ Once weekly ☐ Twice weekly

☐ thrice weekly ☐ Others.................

N.4.7 Do you observe any molds in the house

☐ Yes, through out the year ☐ Yes, during rainy season only ☐ No

N.5 Kitchen Characteristics

N.5.1 What type of kitchen do you have?

☐ Separate indoor ☐ Indoor with Partition

☐ Indoor without Partition ☐ Separate Outdoor

N.5.2 How often do you usually cook for yourself or your house hold? (tick one)

☐ Less than once a month ☐ 1-3 times per month

☐ 1-3 times per week ☐ 4-6 times per week ☐ Every day

N.5.3 On days when you cook, how many hours per day do you typically spend cooking? (tickone)

☐ < 1 hr ☐ 1-2 hrs ☐ 2-3hrs ☐ > 3hrs

N.5.4 What type of fire/stove is used in your household for cooking? (tick all that apply but star the one that is used most often)

☐ LPGstove ☐ Electric stove ☐ Traditional clay

☐ Improved traditional ☐ Other (Specify)

N.5.5 What type of fuel is used in your house hold for cooking?

☐ LPG ☐ Electricity ☐ Kerosene ☐ Coal/Coke

☐ Firewood ☐ Other (Specify)

N.5.6 If you cook indoors, does the room you cook in have any of the following? (tick all that apply)

☐ Windows that open ☐ Chimney ☐ Extractor fan

☐ Other form of ventilation (specify)

N.5.7 When you cook, do you have a door or window to the outside air open or you operate an exhaust fan?

☐ Yes ☐ No ☐ Do not have window/exhaust

N.5.8 Which of the following are used to heat water in this home?

☐ LPG ☐ Electricity (Geysers/Electricrod)

☐ Kerosene ☐ Coal/Coke ☐ Firewood

N.5.9 If it is cold which of the following methods do you use to heat your living room?

☐ Don't heat ☐ Electric heaters ☐ Coal/Coke ☐ Firewood

N.6 Time Activity

N.6.1 How much time does your kid spend in school?

☐ 5hr ☐ 6hrs ☐ 7hrs ☐ >7 hrs

N.6.2 How much time does your kid take to commute to school (to and fro)?

☐ 30mins ☐ 30-60 mins ☐ 60-90 mins ☐ 90-120 mins ☐ > 120mins

N.6.3 What is typical mode of commute of your kid to the school?

☐ Bus ☐ Auto ☐ Bicycle ☐ Car

☐ Metro/Train ☐ Motorbikes ☐ Walking

N.6.4 How much time (in mins) typically does your kid spend outside home daily?

☐ 30 mins ☐ 30-60mins

☐ 60-90 mins ☐ 90-120mins ☐ > 120 mins
