# Supplementary material for: Rationale and methodology of a multicentric prospective cohort study on ‘Longitudinal Effects of Air Pollution Exposure on Adolescent Lungs (APEAL)’ in urban India: APEAL protocol
Source: BMJ Open. 2025 Aug 12;15(8):e106329. doi: 10.1136/bmjopen-2025-106329 (PMC12352163; doi:10.1136/bmjopen-2025-106329)
Supplement: online supplemental file 2 [file bmjopen-15-8-s002.docx]

**Supplemental material 2**

**Standard operating procedures for sample collection: APEAL Study**

***Blood sample***

A blood sample (5ml) will be collected at baseline by venipuncture in the presence of the parent at room temperature. The collected samples will be centrifuged at the end of the clotting time (30-60 minutes) in a horizontal rotor (swing-out head) for 20 minutes at 2200 RPM at room temperature. The separated serum is to be stored at -80^o^C in three aliquots of 1ml each. The samples will be collected again in the fourth year (V4). These blood samples will be used to determine blood levels of ‘early’ blood biomarkers to predict lung injury and lung function deficit in susceptible population.

***Hair and nail sample***

Hair samples will becollected from the neck region about 1-2 cm away from the scalp, near the nape using sanitized round edged stainless-steel scissors. About 4 – 5 g of the sample is to be collected from each individual and stored in a closed, sterile, polyethylene zip lock labelled bag until extraction. Nail plate samples as close to the hyponychium as safely possible (about 2-3 mm length from each fingernail, total 2-3 g of sample) is to be collected using sterile clippers, and then stored in a closed, sterile, polyethylene labelled bag until extraction. To ensure that sufficient mass is collected, toenails/fingernails from all ten fingers will be collected. After cutting, the respondent will be asked to put the sample directly into the polyethylene zip lock bag, already labelled by the researcher. The samples will be collected again in the fourth year (V4) apart from the baseline (V1). These samples will be used to quantify toxic heavy metals to understand the true exposure and dose of air pollutants in children.
