# Supplementary material for: Rationale and methodology of a multicentric prospective cohort study on ‘Longitudinal Effects of Air Pollution Exposure on Adolescent Lungs (APEAL)’ in urban India: APEAL protocol
Source: BMJ Open. 2025 Aug 12;15(8):e106329. doi: 10.1136/bmjopen-2025-106329 (PMC12352163; doi:10.1136/bmjopen-2025-106329)
Supplement: online supplemental file 3 [file bmjopen-15-8-s003.docx]

**Supplemental material 3**

**Lung function tests: Methods and Equipment**

***Spirometry***

We are using the Easy one Air spirometry machine (portable handheld device) to perform pre and post bronchodilator test on all the recruited participants at baseline and will be repeated in all participants every year till four years. If children are unable to do the spirometry even after 3 attempts, they are to be assessed for the rest of the parameters. Standard ATS/ERS guidelines will be used for interpretation.

***Forced Oscillation Technique (FOT) or Impulse Oscillometry***

FOT is an effort - free lung assessment method that measures airway obstruction. It is a clinically proven alternative method for testing lung function using simple tidal breathing and is independent of patient effort. It is particularly suited for elderly and children, requires little training. FOT works on the principle of forced oscillation where the device sends multi-frequency pressure waves in the range 5-30Hz super-imposed on tidal breathing. By measuring the airflow and pressure, the device calculates respiratory impedance, a measure of obstruction.

Pulmoscan is a portable, stand alone, wireless Forced Oscillation Technique (FOT) device^29^. We also used this machine also in our study to measure airway obstruction. This technique measures airway Impedance which is a combination of airway resistance and airway reactance. Resistance at 5Hz (R5), Reactance at 5 Hz (X5) and Difference of resistance at 5 and 20 Hz (R5-R20) will be observed for change in patterns. A pre and post bronchodilator FOT test will be done among all the participants. This test will be done at baseline and repeated every year till next four years.
