# Supplementary material for: Rationale and methodology of a multicentric prospective cohort study on ‘Longitudinal Effects of Air Pollution Exposure on Adolescent Lungs (APEAL)’ in urban India: APEAL protocol
Source: BMJ Open. 2025 Aug 12;15(8):e106329. doi: 10.1136/bmjopen-2025-106329 (PMC12352163; doi:10.1136/bmjopen-2025-106329)
Supplement: Supplementary file 4 [file bmjopen-2025-106329.reviewer_comments.pdf]

# PEER REVIEW HISTORY

BMJ Open publishes all reviews undertaken for accepted manuscripts. Reviewers are asked to complete a checklist review form and are provided with free text boxes to elaborate on their assessment. These free text comments are reproduced below.

## ARTICLE DETAILS

### Title (Provisional)

APEAL protocol: rationale and methodology of a multicentric prospective cohort study on “Longitudinal Effects of Air Pollution Exposure on Adolescent Lungs (APEAL)” in urban India

### Authors

Agarwal, Twinkle; Phuleria, Harish C; Mohan, Anant; D'Souza, George; Thimmulappa, Rajesh; B.S, jayaraj; Mani, Minu Rose; Patil, Swapnali; Samdarshi, Priya; Nori-Sarma, Amruta; Wellenius, Gregory; Mahesh, Padukudru Anand

---

This article was not externally reviewed at BMJ Open. Protocols that have been independently assessed prior to submission to BMJ Open are usually fast-tracked to publication on the grounds that further substantial changes will not be possible. This independent assessment will usually be external, independent review for both a substantial grant award from a non-commercial or government funder and ethics approval.
